# Osteoarthritis: multitissue pathology, molecular mechanisms, clinical management, and emerging precision and regenerative therapies

**DOI:** 10.3389/fphar.2025.1697192

**Published:** 2026-01-05

**Authors:** Lina Qiu, Ahmad Alhaskawi, Safwat Adel Abdo Moqbel

**Affiliations:** 1 Nursing Department, The Second Affiliated Hospital of Zhejiang University School of Medicine, Hangzhou, China; 2 Department of Orthopedics, the First Affiliated Hospital of Zhejiang University School of Medicine, Hangzhou, China; 3 Department of Emergency, the Second Affiliated Hospital of Zhejiang University School of Medicine, Hangzhou, China

**Keywords:** osteoarthritis, chondrocyte ferroptosis, synovial inflammation, regenerative therapy, meniscus degeneration

## Abstract

Osteoarthritis (OA) is a progressive, whole-joint disorder driven by a convergence of biomechanical stress, inflammation, metabolic dysfunction, and cellular senescence. This review integrates recent advances in our understanding of the distinct yet interconnected pathological processes affecting articular cartilage, subchondral bone, synovium, infrapatellar fat pad, menisci, ligaments, and peri-articular musculature. Emerging mechanisms, such as chondrocyte ferroptosis, neurovascular remodeling, and synovial-mesenchymal reprogramming, are highlighted for their roles in disease propagation and chronic pain. We critically appraise current therapeutic modalities, including evidence-based non-pharmacological strategies, pharmacologic agents, intra-articular biologics, and surgical interventions. In parallel, we explore the promise of precision medicine, multi-omics profiling, advanced imaging biomarkers, regenerative therapies, and artificial intelligence in reshaping diagnostic and treatment paradigms. This comprehensive synthesis underscores the shift toward a mechanistic, individualized approach to OA management and identifies key translational opportunities for disease modification and early intervention.

## 1 Introduction

**FIGURE 1:**
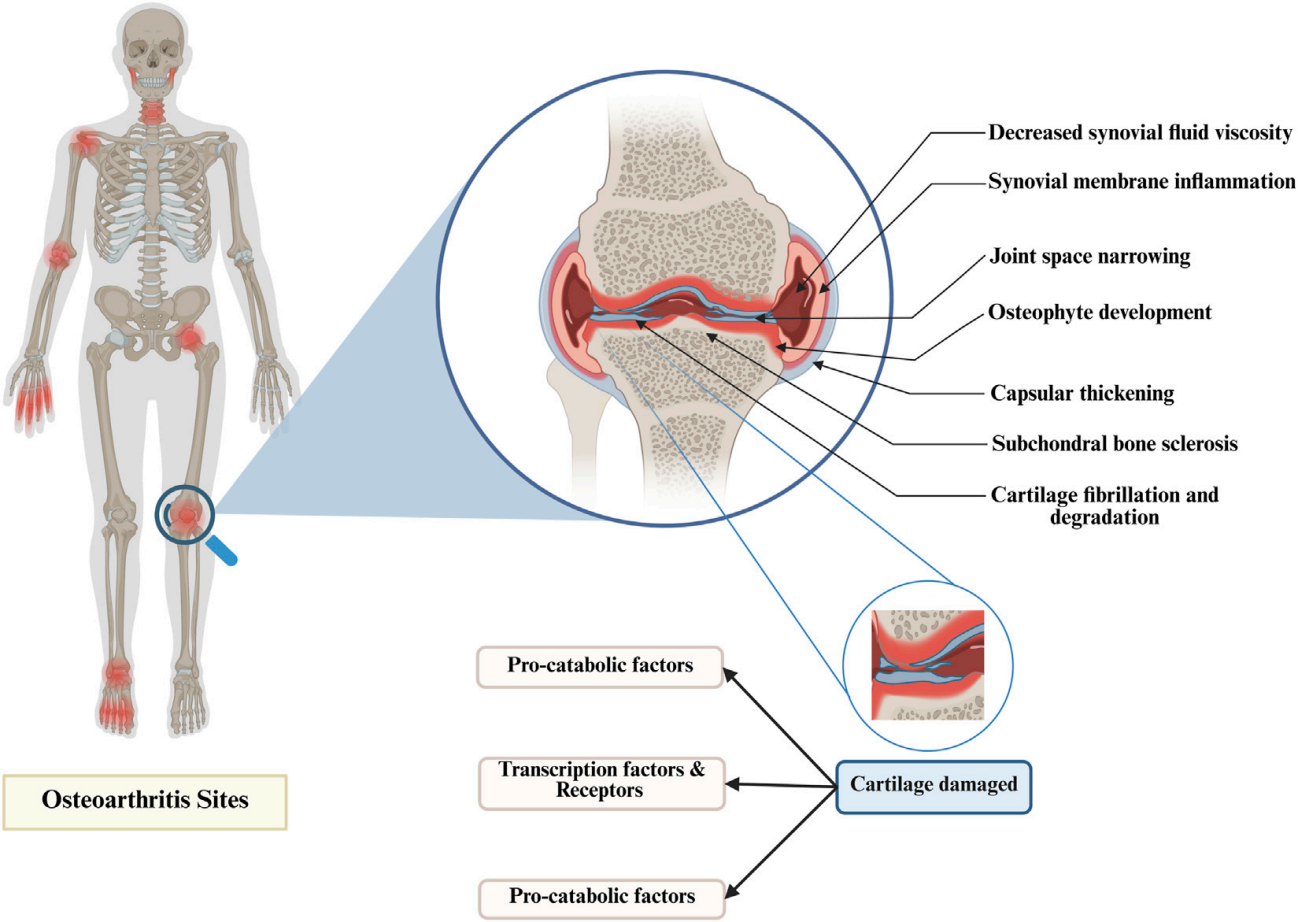
Structural joint changes in osteoarthritis.

**FIGURE 2:**
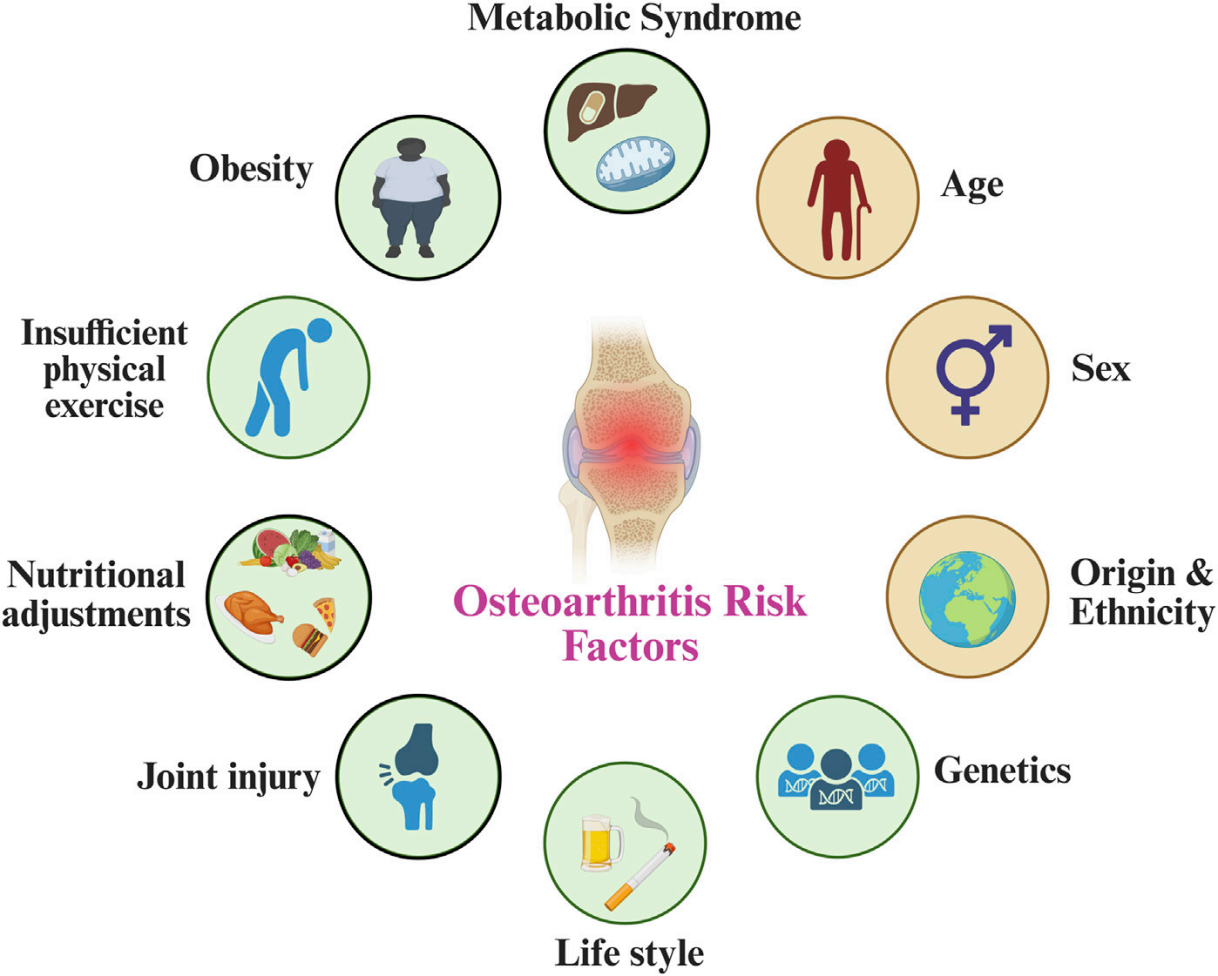
Overview of the common key risk factors contributing to osteoarthritis.

Osteoarthritis (OA) is a prevalent and debilitating musculoskeletal disorder that affects the structural and functional integrity of synovial joints. Globally, OA represents a substantial public health concern, impacting over 500 million individuals and contributing significantly to reduced quality of life, work loss, and healthcare expenditure (Ma W. et al., 2025; Leifer et al., 2022). No longer regarded merely as a consequence of mechanical wear and tear, OA is now conceptualized as a complex, whole-joint disease characterized by progressive degeneration of articular cartilage, subchondral bone remodeling, synovial inflammation, and alterations in surrounding peri-articular structures (Di Nicola, 2020; Coaccioli et al., 2022) (Figure 1). This multifactorial condition is driven by a convergence of biomechanical, inflammatory, metabolic, and post-traumatic processes, which collectively compromise joint homeostasis and lead to pain, stiffness, and functional disability. In addition, the disease disproportionately affects weight-bearing joints, particularly the knees and hips, and its incidence continues to rise in parallel with population aging and increasing prevalence of obesity. Notably, obesity and joint injury remain the most prominent modifiable risk factors, whereas age, sex, and genetic predisposition are considered non-modifiable contributors (Figure 2) (Nedunchezhiyan et al., 2022; Arden and Nevitt, 2006). Current management strategies emphasize symptom relief through non-pharmacological approaches such as exercise, education, and weight management. However, adherence to these interventions remains suboptimal, limiting their long-term effectiveness (Farinelli et al., 2024; Anandacoomarasamy and March, 2010). For individuals with advanced disease and persistent symptoms refractory to conservative treatment, joint replacement surgery is a viable option (Anandacoomarasamy and March, 2010). Despite ongoing advances in our understanding of OA pathogenesis, there remains an urgent need to develop disease-modifying therapies and improve early intervention strategies to mitigate the escalating global burden of OA.

In this review, we integrate recent advances in the understanding of osteoarthritis pathophysiology, spanning cartilage, subchondral bone, synovium, and peri-articular tissues, to highlight their interconnected roles in disease initiation and progression. We further examine emerging molecular mechanisms, including ferroptosis, synovial reprogramming, and neurovascular remodeling, and critically evaluate current and prospective therapeutic strategies. By bridging basic science with clinical application, this review aims to provide a comprehensive, multidisciplinary perspective on the evolving landscape of OA research, diagnosis, and management.

## 2 Pathological changes associated with osteoarthritis

### 2.1 Subchondral bone

Subchondral bone plays a critical role in the pathogenesis and progression of OA, undergoing a series of pathological alterations that contribute to joint degeneration and pain (Zhu et al., 2020). In the early stage of OA, the subchondral bone exhibits increased remodeling activity, characterized by elevated bone turnover, microstructural deterioration, and a shift from bone resorption to formation. These changes lead to sclerosis, trabecular thickening, and reduced mechanical shock absorption, which exacerbates cartilage degradation due to increased mechanical stress on the overlying articular surface (Zhu et al., 2020; Li et al., 2013). One hallmark feature of OA-related subchondral bone pathology in early stages is the disruption of its architecture, including the formation of bone marrow lesions (BMLs), microcracks, diffuse microdamage, and marginal osteophytes. These abnormalities are often associated with pathological angiogenesis and nerve invasion, contributing to inflammation and nociception (Li et al., 2013; Felson, 2004). Notably, subchondral nerve ingrowth, often occurring alongside neovascularization, introduces sensory and sympathetic nerve fibers into previously aneural regions of bone. This aberrant innervation sensitizes the subchondral microenvironment, contributing to the chronic pain experienced in OA (Morgan et al., 2022). Nerve growth factor (NGF), released by osteoblasts, endothelial cells, and infiltrating immune cells, plays a key role in promoting this ectopic innervation, establishing a direct link between structural bone changes and pain signaling (Grässel and Muschter, 2017). In addition, osteoclast promotes axonal growth by secreting axon-guiding molecules such as netrin-1. Netrin-1, acting *via* its receptor DCC, has been shown to drive the sprouting of calcitonin gene-related peptide (CGRP)+ nociceptive fibers in the subchondral bone, thereby contributing directly to pain hypersensitivity associated with OA (Zhu et al., 2019). Osteophytes, though anatomically distinct from subchondral bone, are closely associated with its pathological changes in OA. These osteocartilaginous outgrowths form at the joint margins at the interface of the synovium, periosteum, and articular cartilage, yet their development is tightly linked to the mechanical and molecular alterations occurring in the subchondral region (Li et al., 2013). They are formed through an endochondral-like process involving progenitor cells of the Gdf5 lineage (Wong et al., 2016). These include Sox9-expressing cells in the periosteum, which generate hybrid skeletal cells, and Prg4-expressing synovial cells that contribute chondrocytes to the cartilage cap of the osteophyte (Roelofs et al., 2020). While initially stabilizing the joint, osteophytes may disrupt biomechanics, impinge on adjacent tissues, and contribute to pain and dysfunction in OA. Histopathologically, several features distinguish OA subchondral bone from healthy bone. These include the presence of BMLs, fibrosis, microfractures, and abnormal vascularization, which strongly correlate with pain severity and structural disease progression (Aho et al., 2017). Microcracks and diffuse microdamage are frequently observed in calcified cartilage and adjacent trabecular bone, contributing to localized remodeling and osteocyte dysfunction (Seref-Ferlengez et al., 2015). At the cellular level, early OA subchondral bone remodeling is driven by an imbalance between bone-resorbing osteoclasts and bone-forming osteoblasts. Osteoclastogenesis is upregulated due to increased expression of receptor activator of nuclear factor kappa-B ligand (RANKL) and cathepsin K, resulting in enhanced bone resorption (Chen W. et al., 2024; Henrotin et al., 2012). Meanwhile, osteoblasts in OA exhibit a pathological phenotype, marked by increased secretion of RANKL, VEGF, and TGF-β1, and impaired mineralization due to altered collagen synthesis. The accumulation of osteoid tissue with poor mineral quality contributes to hypomineralization and bone fragility (Table 1) (Maruotti et al., 2017). Additionally, osteocytes display impaired perilacunar/canalicular remodeling and diminished mechanosensing capacity, further disrupting local bone homeostasis (Zhu et al., 2020; Donell, 2019). In the late stage of OA, subchondral bone shows advanced structural damage. Prominent features include subchondral bone cysts, also known as intraosseous lesions, that form in regions subjected to repetitive mechanical overload. These cysts are characterized by osteoclastic resorption at their periphery and poorly mineralized new bone formation internally, reflecting highly dysregulated turnover and structural compromise (Feng and McDonald, 2011; Ott, 2008). Other features include necrotic marrow, fibrosis, and persistent abnormal vascularization, all of which correlate with disease severity and increased joint pain. Cellular dysfunction continues in the late stage, with a persistent imbalance between osteoclast and osteoblast activity. Osteoblasts continue producing excessive osteoid with poor mineral quality, contributing to bone fragility and structural weakness (Maruotti et al., 2017). Osteocyte dysfunction remains a key contributor to abnormal bone remodeling in advanced disease (Zhu et al., 2020; Donell, 2019).

**TABLE 1:** Molecular pathology across joint tissue in osteoarthritis.

Tissue	Key molecular and signaling changes	Pathological consequences
Articular Cartilage	↑ MMP-13, ADAMTS-5 → ECM degradation ↓ SOX9, COL2A1, ACAN → Reduced matrix synthesis ↑ RUNX2, COL10A1, Wnt/β-catenin, NF-κB → Chondrocyte hypertrophy and inflammation	Fibrillation, softening, surface erosion Chondrocyte hypertrophy and apoptosis
Subchondral Bone	↑ TGF-β1, BMP-2, VEGF → Aberrant remodeling and angiogenesis ↑ RANKL/OPG imbalance → Osteoclastogenesis ↑ DKK1, SOST → Wnt signaling suppression	Sclerosis, bone marrow lesions Osteophyte formation Structural deformation
Synovium	↑ IL-1β, TNF-α, IL-6, IL-17 → Inflammation ↑ COX-2, PGE2, MMP-1/3, iNOS → Matrix degradation and vasodilation ↑ CD68^+^ macrophages, Th17 infiltration	Synovitis Pain, swelling Promotion of cartilage breakdown
Ligaments	↑ IL-6, MMP-2, MMP-13 → ECM remodeling ↓ Tenomodulin, SCX, TNMD → Impaired ligament homeostasis ↑ NF-κB, TGF-β1 signaling	Ligament laxity and degeneration Instability contributing to joint misalignment
Meniscus	↑ MMP-1, MMP-3, ADAMTS-4/5 → ECM breakdown ↑ IL-1β, TNF-α, HMGB1 → Inflammation and catabolism ↓ ACAN, COL2A1	Meniscal extrusion, tears Reduced shock absorption Accelerated OA progression
Peri-articular Muscles	↑ TNF-α, IL-6, myostatin → Muscle atrophy ↓ IGF-1, Akt/mTOR pathway activity → Impaired regeneration ↑ oxidative stress, autophagy markers (LC3-II, Beclin-1)	Muscle weakness Reduced joint support Functional decline and increased OA severity

Recent studies have also highlighted the molecular mechanisms underlying these pathological changes. Dysregulated signaling pathways such as Wnt/β-catenin, TGF-β, and PI3K/Akt have been shown to modulate osteoblast and osteoclast activity in OA subchondral bone. Furthermore, subchondral angiogenesis, often driven by VEGF and mechanical stress, disrupts the osteochondral interface and promotes cartilage deterioration (Grässel et al., 2021; Noh et al., 2020).

### 2.2 Synovium and infrapatellar fat pad

In OA, the synovial membrane exhibits marked pathological remodeling characterized by lining layer hyperplasia, sublining fibrosis, increased vascularization, and infiltration of immune cells, particularly macrophages, lymphocytes, and mast cells. Fibroblast-like synoviocytes (FLS) in the intimal layer adopt a pro-inflammatory phenotype under chronic stimulation, secreting cytokines such as IL-1β, TNF-α, IL-6, and chemokines that attract monocytes and perpetuate synovitis. This altered microenvironment enhances production of matrix-degrading enzymes (e.g., MMPs, ADAMTS), which degrade cartilage matrix and disrupt joint homeostasis (Sanchez-Lopez et al., 2022; Scanzello and Goldring, 2012). Moreover, tissue-resident type A synoviocytes (macrophage-like) are key mediators of innate immune responses and interact with FLSs to potentiate inflammatory cascades, further contributing to cartilage destruction and joint pain. Synovial inflammation is closely associated with peripheral sensitization *via* nerve growth factor (NGF) signaling, and imaging studies reveal strong correlations between synovial thickening, effusion, and OA symptom severity (Panichi et al., 2024; Bai et al., 2022). A comprehensive bioinformatics analysis review revealed 143 differentially expressed genes in OA synovial tissue, primarily involved in immune response, inflammation, and chemokine signaling pathways. Using protein-protein interaction networks, key hub genes, including IL6, CXCL8, and IL1B, were identified as central to OA pathogenesis (Zou et al., 2023). Lin et al. identified a distinct subset of OA-associated macrophages (OA-macrophages) that exhibit heightened pro-inflammatory and pro-fibrotic activity in synovial and infrapatellar fat pad, contributing significantly to leukocyte recruitment, chondrocyte apoptosis, and angiogenesis. They further developed a diagnostic model, OAMGS, based on four marker genes (VEGFA, CDKN1A, PIM3, MAFF) using machine learning, validated in both human and rat models (Lin et al., 2025). In addition, Huang et al. explored synovial cell heterogeneity and demonstrated the feasibility of computationally deconvolving bulk RNA-seq data into single-cell resolution using customized gene signature matrices, especially *via* CIBERSORTx. They identified seven major synovial cell types and emphasized the underestimated role of T cells and macrophage subtypes in OA pathogenesis (Huang Z. Y. et al., 2022).

**FIGURE 3:**
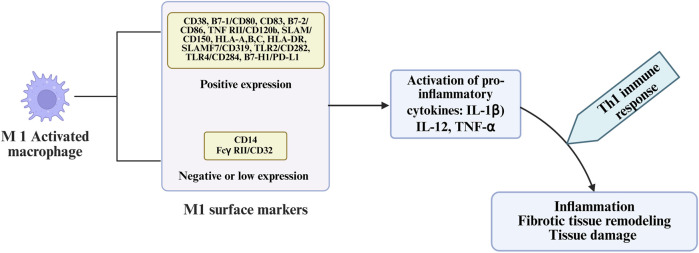
M1 macrophage activation, characterized by specific surface marker expression and secretion of pro-inflammatory cytokines.

The infrapatellar fat pad (IFP), or Hoffa’s fat pad, is an intra-articular but extrasynovial structure rich in adipocytes, immune cells, blood vessels, and nerve fibers (Braun et al., 2022). In OA, the IFP undergoes significant morphological and molecular alterations, including adipocyte hypertrophy, fibrosis of interlobular septa, neovascularization, and infiltration by pro-inflammatory immune cells, particularly M1-polarized macrophages (Figure 3). This tissue becomes a potent source of adipokines (e.g., leptin, resistin, visfatin), cytokines, and prostaglandins that act locally and systemically to exacerbate joint inflammation, cartilage catabolism, and pain perception (Braun et al., 2022; Wang M. G. et al., 2024). Recent evidence has demonstrated that aging and obesity drive maladaptive remodeling of the IFP, characterized by chronic low-grade inflammation, accumulation of senescent cells, and expression of senescence-associated secretory phenotype (SASP) factors, such as MMP-13, IL-1β, and RANTES. These mediators promote extracellular matrix remodeling, vascular activation, and further immune recruitment, creating a self-sustaining inflammatory niche that facilitates OA progression (Wang B. et al., 2024; Musale et al., 2023). Emerging molecular and histological evidence suggests that the IFP and synovium form a single anatomo-functional entity. Single-cell RNA-sequencing has revealed that both tissues originate from shared mesenchymal progenitor cells, particularly Dpp4+ stromal populations, which give rise to synovial fibroblasts, adipocytes, and myofibroblasts under OA conditions. These progenitors exhibit pro-fibrotic and pro-inflammatory differentiation trajectories in response to joint injury. Coordinated pathological changes include fibrosis, stromal stiffening, and myofibroblast expansion in both compartments, which compromise joint biomechanics and serve as sources of persistent nociceptive signaling (Tang et al., 2024; Li et al., 2024). Furthermore, the IFP and synovium serve as reservoirs of mesenchymal stromal cells (MSCs) with potential immunomodulatory functions. However, under OA conditions, these MSCs are reprogrammed toward pro-inflammatory phenotypes, losing their reparative capacity and instead contributing to disease propagation. Fibrosis alters IFP biomechanics, reducing its cushioning capacity and increasing mechanical stress on adjacent joint structures, thereby accelerating cartilage degeneration (Jeyaraman et al., 2022; Garcia et al., 2016; Zupan et al., 2020).

### 2.3 Cartilage

Cartilage in healthy joints maintains a precise balance between synthesis and degradation of ECM components, primarily type II collagen and aggrecan, orchestrated by chondrocytes. In OA, this balance is disrupted by catabolic signaling, inflammation, and mechanical stress, culminating in progressive cartilage erosion and exposure of subchondral bone (Peng Z. et al., 2021). The onset of OA pathology in cartilage is marked by an early shift in chondrocyte phenotype from a homeostatic to a catabolic and hypertrophic state. Pro-inflammatory cytokines, particularly IL-1β, TNF-α, and IL-6, are key mediators that stimulate chondrocytes to upregulate degradative enzymes such as matrix metalloproteinases (MMP-1, MMP-3, MMP-13) and aggrecanases (ADAMTS-4 and ADAMTS-5). These enzymes target and cleave aggrecan and collagen fibrils, leading to loss of tensile strength, increased water content, and impaired load-bearing capacity of the matrix (Yunus et al., 2020; Charlier et al., 2019). At the same time, anabolic signaling pathways are suppressed, with downregulation of COL2A1 and ACAN expression (Table 1). These events are compounded by oxidative stress and mitochondrial dysfunction, which contribute to DNA damage, chondrocyte senescence, and impaired autophagy. Reactive oxygen species (ROS) exacerbate matrix degradation and promote further inflammation, establishing a self-sustaining loop of cartilage damage (Lepetsos and Papavassiliou, 2016; Hong et al., 2024). A pivotal early event in OA cartilage pathology is the degradation of the pericellular matrix (PCM), which surrounds chondrocytes and facilitates mechanotransduction. As shown by high-resolution imaging and proteomic analyses, PCM damage impairs cellular responses to mechanical stimuli and nutrient diffusion, leading to chondrocyte death and clustering. These clusters are typically observed in the middle and deep zones of the cartilage and are indicative of attempted, but dysregulated, repair responses. The loss of PCM integrity also allows deeper penetration of cytokines and enzymes, accelerating matrix breakdown (Goldring, 2012; Umlauf et al., 2010). The phenotypic transformation of chondrocytes toward a hypertrophic state is evidenced by upregulation of type X collagen (COL10A1), alkaline phosphatase (ALP), and VEGF. This hypertrophic shift recapitulates features of endochondral ossification and contributes to cartilage calcification, tidemark duplication, and eventual osteophyte formation at joint margins (Rim et al., 2020). Moreover, chondrocyte apoptosis, triggered by ER stress, nitric oxide, and oxidative insults, plays a critical role in cartilage degradation. Apoptotic bodies and released damage-associated molecular patterns (DAMPs) further stimulate inflammatory signaling in surrounding tissues, promoting joint-wide degeneration (Guan et al., 2024; Takada et al., 2011). Furthermore, chondrocyte ferroptosis is a type of regulated cell death driven by the accumulation of lipid peroxides and disruption of redox homeostasis, which severely affects cartilage integrity (Yao et al., 2021). In chondrocytes, ferroptosis occurs primarily due to oxidative stress, iron overload, and impaired antioxidant defense mechanisms such as reduced activity of glutathione peroxidase 4 (GPX4) and the cystine/glutamate antiporter system Xc. These disruptions lead to excessive lipid ROS accumulation, resulting in cellular damage and matrix degradation. Inflammatory stimuli further aggravate ferroptosis in chondrocytes, contributing to pathological changes in the cartilage environment (Yao et al., 2021; Fan et al., 2024). The JNK-JUN-NCOA4 pathway has been identified as a critical regulator, promoting iron accumulation *via* ferritinophagy (Sun et al., 2023). Protective factors such as Nrf2 and SLC7A11/GPX4 can counteract this process by reducing oxidative damage (Wan et al., 2023). Li et al. combined single-cell RNA sequencing and population-level genetic analyses to reveal two key chondrocyte subpopulations, inflammatory and fibrocartilage chondrocytes, that are expanded in hand OA and enriched for ferroptosis-related and inflammatory pathways, with elevated expression of FTH1, implicating iron overload in OA pathogenesis (Li H. et al., 2023). Supporting this, Sun et al. identified CD8^+^ T cells in the synovium as drivers of cartilage degeneration and subchondral bone damage through interactions with chondrocytes, offering mechanistic insight into immune-mediated OA progression (Sun et al., 2024). Nevertheless, chondrocyte senescence refers to the irreversible growth arrest of cartilage cells, characterized by altered gene expression, impaired matrix production, and secretion of pro-inflammatory and degradative factors collectively known as the senescence-associated secretory phenotype (SASP) (Loeser, 2009). This process is primarily triggered by cumulative oxidative stress, DNA damage, and telomere attrition, which activate key pathways such as p53/p21 and p16^INK4a/Rb, leading to permanent cell cycle arrest (Loeser, 2009). Mechanical stress and mitochondrial dysfunction also contribute significantly to the induction of senescence in chondrocytes, disrupting their homeostasis and regeneration potential (Jiang et al., 2023). Furthermore, the overexpression of caveolin-1 has been associated with promoting senescent phenotypes, indicating its involvement in age-related cartilage degeneration (Aigner et al., 2007). These mechanisms collectively lead to the progressive deterioration of cartilage, establishing senescence as a central player in joint aging and osteoarthritis pathogenesis. OA disrupts the natural zonal architecture of cartilage. The superficial zone, typically rich in lubricin (PRG4) and oriented collagen fibers, is eroded early, compromising lubrication and tensile resistance. The middle zone, responsible for absorbing compressive forces, becomes disorganized as proteoglycan loss advances. In the deep zone, vertical collagen bundles are fragmented, and the calcified cartilage zone expands abnormally, with advanced mineral deposition leading to stiffening and altered load transmission across the osteochondral unit (Sophia Fox et al., 2009; Eschweiler et al., 2021).

On the other hand, the bidirectional crosstalk between synovium and articular cartilage amplifies inflammation and matrix degradation across the whole joint. In OA, fibroblast-like synoviocytes and synovial macrophages acquire a pro-inflammatory phenotype, secreting high levels of cytokines and chemokines and prostaglandins, together with matrix-degrading enzymes and nitric oxide. These mediators diffuse through the synovial fluid to the cartilage surface, where they drive chondrocytes toward catabolic, hypertrophic, or senescent phenotypes, upregulate MMPs and aggrecanases, suppress COL2A1 and ACAN expression, and promote oxidative stress and ferroptosis, thereby accelerating ECM breakdown (Chou et al., 2020; Wang et al., 2022). Conversely, stressed or dying chondrocytes release DAMPs, apoptotic bodies, cartilage fragments, and pro-inflammatory mediators that further activate synovial macrophages and fibroblasts, maintaining a chronic synovitic state and perpetuating pain and swelling (Fan et al., 2024; Li and Sun, 2024). In addition to soluble cytokines, synovium-cartilage communication is increasingly recognized to be mediated by extracellular vesicles and exosomes carrying microRNAs, long non-coding RNAs, and bioactive proteins (Wessler and Meisner-Kober, 2025; Asghar et al., 2020).

### 2.4 Ligaments, meniscus, and peri-articular muscles

In OA, ligaments, especially the cruciate (ACL/PCL) and collateral ligaments, undergo both structural and biochemical deterioration. Histological studies have demonstrated disorganization of collagen fiber alignment, increased vascularity, mucoid degeneration, and loss of fibroblast density (Cho et al., 2012). Cruciate ligaments often show dystrophic mineralization and partial ruptures in advanced disease stages, particularly in knees with varus or valgus malalignment. Molecularly, ligament fibroblasts in OA upregulate MMP and inflammatory mediators, contributing to ECM degradation and mechanical instability (Table 1) (Nyland et al., 2020). Aging and OA synergistically affect the mechanical properties of ligaments, reducing tensile strength and altering viscoelasticity, thereby impairing joint proprioception and load distribution. These ligamentous alterations also predispose to further structural deterioration in the meniscus and cartilage (Peters et al., 2018; Loeser, 2017). The meniscus, a fibrocartilaginous structure essential for load transmission, shock absorption, and joint congruency, exhibits significant degenerative changes in OA. Macroscopically, OA-associated meniscal pathology includes partial maceration, radial and horizontal tears, and extrusion from the joint line. MRI studies frequently reveal meniscal degeneration in early OA, often preceding radiographic evidence of cartilage loss (Makris et al., 2011; Melrose, 2019). Histopathologically, OA menisci exhibit surface fibrillation, collagen disorganization, hypocellularity, calcification, and formation of fibrocartilaginous metaplasia. These changes compromise the biomechanical function of the meniscus and reduce its capacity to distribute load, thereby increasing stress on adjacent cartilage and subchondral bone. Meniscal extrusion has been associated with accelerated medial joint space narrowing and correlates strongly with knee pain and OA progression (Langhans et al., 2023; Ozeki et al., 2022). Emerging evidence also suggests that meniscal cells actively contribute to the inflammatory microenvironment. Degenerated menisci produce catabolic mediators such as prostaglandin E2 (PGE2), nitric oxide, and cytokines that can diffuse into synovial fluid and exacerbate articular cartilage degeneration (Krupkova et al., 2018). In addition, meniscectomy, whether partial or total, significantly contributes to the development and progression of OA through structural and biomechanical alterations in the knee joint. Studies have shown that total meniscectomy leads to a substantially higher risk of OA progression compared to partial meniscectomy, with increased incidence of joint space narrowing and osteophyte formation (Ma X. et al., 2025; Cabarcas et al., 2025; Migliorini et al., 2023). Moreover, partial meniscectomy, though considered less invasive, still results in long-term joint degeneration, especially when combined with factors such as limb malalignment or ACL deficiency (Zhang H. et al., 2025; Wechsler and Rotem, 2025). Histopathological evaluations demonstrate increased cartilage fibrillation, matrix loss, and inflammatory cytokine expression post-meniscectomy (Kwok et al., 2016; Haemer et al., 2011). Thus, meniscal preservation remains critical in delaying OA onset, underscoring the importance of repair technique over excision whenever possible. Furtheoremore, Varus and valgus alignments alter load-bearing dynamics across the tibiofemoral joint, leading to compartment-specific degeneration. Varus alignment, often associated with medial compartment OA, increases compressive forces on the medial meniscus and cartilage, accelerating cartilage breakdown and osteophyte formation (Valente et al., 2025; Terauchi et al., 2025). Conversely, valgus morphotype predisposes the lateral compartment to degenerative changes, especially in conjunction with lateral meniscal damage (Slynarski et al., 2025). Recent gait and imaging analyses further support that morphological features such as tibial plateau slope, femoral condyle shape, and joint congruency significantly affect joint biomechanics, contributing to early onset OA in at-risk individuals (Zhang K. et al., 2025). Therefore, understanding and assessing individual knee morphology is vital in predicting OA risk and optimizing preventive or surgical strategies.

Furthermore, peri-articular muscle weakness and atrophy are common but under-recognized features of OA. Quadriceps and hamstring muscle mass and strength are often significantly reduced in patients with symptomatic knee OA. This sarcopenia-like phenotype arises from a combination of disuse, altered neuromuscular recruitment patterns, systemic inflammation, and age-related muscle loss (Kim et al., 2023; Shorter et al., 2019). Peri-articular muscle biopsies in OA demonstrate fiber-type atrophy (especially type II fast-twitch fibers), increased infiltration by adipocytes and macrophages, fibrosis, and mitochondrial abnormalities. Inflammatory cytokines such as IL-6 and TNF-α have been implicated in muscle catabolism and impaired regeneration. These changes impair dynamic joint stabilization and contribute to further joint instability and pain, forming a deleterious feedback loop with intra-articular degeneration (Table 1) (Cruz Ayala et al., 2022; Terracciano et al., 2013). Quantitative MRI and ultrasound have confirmed reductions in muscle cross-sectional area and quality (e.g., increased fat infiltration), even in early-stage OA. Notably, reduced muscle mass is strongly predictive of radiographic progression and poorer functional outcomes (Huber et al., 2020; Diaz et al., 2025; Guzmán-David et al., 2023).

## 3 Management and treatment of osteoarthritis

### 3.1 Non-pharmacological management

Non-pharmacological interventions form the foundation of contemporary OA management and are universally recommended as first-line therapy across all disease stages and phenotypes. They serve as symptomatic treatments and as disease-modifying strategies aimed at optimizing joint function, minimizing structural deterioration, and reducing long-term pharmacologic and surgical burden (Bierma-Zeinstra et al., 2020). The efficacy of these interventions is maximized when delivered as part of an integrated, patient-centered model of care. A central tenet of non-pharmacological management is patient education, which empowers individuals with knowledge about OA pathophysiology, prognosis, and the value of self-management. Educational programs, particularly those that include shared decision-making and goal-setting components, have been shown to significantly improve treatment adherence and functional outcomes. Patients who understand the biomechanical and inflammatory underpinnings of OA are more likely to engage consistently with lifestyle modifications (Moseng et al., 2024). Rannou et al. highlighted patient education as a cornerstone of OA management. Endorsed by EULAR with level A evidence, education helps patients understand the purpose and benefits of interventions like exercise and weight management. In addition, they emphasized that education should be delivered by both physicians and therapists, and reinforced through follow-ups to sustain engagement and behavioral change (Rannou and Poiraudeau, 2010). Exercise therapy remains the most extensively validated non-pharmacological intervention for OA, supported by numerous randomized controlled trials and systematic reviews. Both aerobic and resistance-based regimens improve pain, enhance mobility, and delay functional decline. Structured programs that combine strength training with proprioceptive and neuromuscular exercises are particularly effective in targeting periarticular muscle weakness, joint instability, and gait abnormalities, hallmarks of lower limb OA (Ambr et al., 2015; Golightly et al., 2012). Supervised physiotherapy may be necessary during the initial stages to ensure safe technique and progressive loading, particularly in frail or deconditioned individuals (Marriott and Birmingham, 2023; Skou and Roos, 2019; Bennell et al., 2014). Conley et al. reviewed multiple high-quality clinical practice guidelines and concluded that exercise, particularly strength training, aerobic activity, and mind-body practices such as tai chi, should be considered a core intervention in OA management, with strong consensus supporting its effectiveness in improving pain and function. They emphasized the importance of individualized, progressive exercise programs tailored to patient needs and capabilities (Conley et al., 2023). Furthermore, Verhagen et al. conducted a meta-epidemiological analysis of 42 randomized controlled trials and demonstrated that exercise yields a clinically meaningful reduction in pain in patients with knee OA, with individually supervised interventions showing greater effectiveness. They concluded that exercise is a well-established, evidence-based treatment for knee OA, making further trials against minimal treatment unnecessary (Verhagen et al., 2019). Closely linked to exercise is weight management, a critical intervention in patients with knee or hip OA who are overweight or obese. Excess body weight increases mechanical load on weight-bearing joints and contributes to systemic inflammation through adipokine dysregulation (Huffman et al., 2024). Clinical evidence demonstrates that a sustained weight loss of ≥10% significantly reduces pain and improves joint function, particularly when combined with physical activity (Wood et al., 2021; Ryan and Yockey, 2017). Behavioral interventions that integrate nutritional counseling with physical rehabilitation have been shown to yield superior outcomes compared to either approach in isolation. Furthermore, physical therapy modalities, including manual therapy, stretching, and functional task training, are frequently incorporated to optimize joint biomechanics and movement quality. For example, interventions targeting hip flexor tightness, quadriceps inhibition, or valgus knee alignment can reduce joint stress and enhance locomotor efficiency. Gait retraining using real-time feedback and corrective bracing may further improve joint load distribution and proprioceptive acuity (Page, 2012; Ni et al., 2024). In patients with biomechanical malalignment or instability, assistive and supportive devices play a complementary role. Off-loading knee braces, lateral wedge insoles, canes, and shock-absorbing footwear can all provide symptomatic relief by modifying joint loading patterns. The prescription of such aids should be tailored by a physiotherapist or orthotist based on a comprehensive biomechanical assessment (Guilak, 2011; Zhang et al., 2020). Finally, the integration of psychosocial interventions, such as cognitive behavioral therapy (CBT) and mindfulness-based stress reduction, addresses the affective dimension of chronic OA pain. Central sensitization, fear-avoidant behavior, and depression are common comorbidities that attenuate treatment response. By enhancing pain coping skills and emotional resilience, these interventions contribute to better functional recovery and health-related quality of life (Zhang et al., 2018; Ordoñez-Mora et al., 2022). Although first-line management strategies for OA are underpinned by robust evidence, including systematic reviews and international guideline recommendations, real-world adherence remains suboptimal. Despite global consensus on the recommended interventions and increasing recognition of the disease’s growing public health burden, many individuals with OA do not receive care aligned with these standards. Furthermore, both healthcare providers and patients demonstrate low levels of engagement with and implementation of these evidence-based protocols (Cunningham et al., 2021; Jönsson et al., 2019; Dziedzic and Allen, 2018). Mazzei et al. reported that although 74% of patients engaged in some form of exercise and 64% received educational support, only 19% adhered to all first-line guideline-consistent treatments, highlighting implementation gaps in clinical settings (Mazzei et al., 2022). Hofstede et al. similarly observed that while many patients received individual interventions (e.g., 80% education, 73% physical therapy), a mere 6% received the full spectrum of recommended therapies, with dietary guidance markedly underutilized, particularly among overweight patients (Hofstede et al., 2015). Cronström et al. further emphasized that only 40% of patients received guideline-based OA management, such as the better management of patients with osteoarthritis program, before being placed on a surgical waitlist, with even fewer receiving such care during the wait, underscoring systemic deficiencies in guideline implementation (Cronström et al., 2020).

### 3.2 Pharmacological management of osteoarthritis

#### 3.2.1 Non-opioid analgesics, anti-inflammatory agents, and senescence/ferroptosis-targeted drugs

Non-opioid analgesics, particularly non-steroidal anti-inflammatory drugs (NSAIDs), are the pharmacological mainstay for symptom control in OA, particularly in cases presenting with moderate to severe pain (Table 2). NSAIDs exert their effect by inhibiting cyclooxygenase (COX) enzymes, thereby reducing the synthesis of prostaglandins involved in pain and inflammation (Williams Benzon et al., 2018; Sohail et al., 2023). Both oral and topical formulations are widely used, with treatment tailored to the patient’s comorbidity profile and risk factors. Oral NSAIDs, such as ibuprofen, naproxen, and celecoxib, have demonstrated consistent efficacy in reducing pain intensity and improving joint function across numerous randomized controlled trials and meta-analyses (Pelletier et al., 2016; Ong et al., 2007). However, their long-term use is constrained by well-documented risks of gastrointestinal, cardiovascular, and renal adverse events. To mitigate these risks, the concurrent use of gastroprotective agents, such as proton pump inhibitors (PPIs), is advised in at-risk populations (Pelletier et al., 2016; Osteoarthritis Group of the Rheumatology and Immunology Physicians Branch of the Chinese Medical Doctor Association, 2024). Moreover, topical NSAIDs, especially topical diclofenac, are strongly recommended as first-line pharmacological therapy for patients with localized OA, particularly of the knee and hand, and in individuals for whom systemic NSAIDs pose unacceptable risk. Their efficacy in providing analgesia is comparable to oral NSAIDs in mild-to-moderate OA, with a substantially lower incidence of systemic side effects (Table 2) (Osteoarthritis Group of the Rheumatology and Immunology Physicians Branch of the Chinese Medical Doctor Association, 2024; Bariguian Revel et al., 2020; Roth and Fuller, 2011). Acetaminophen (paracetamol), once widely used for OA-related pain, is now considered a second-line agent due to its modest analgesic efficacy and potential for hepatotoxicity at higher doses. Current guidelines restrict its role to short-term use in individuals with contraindications to NSAIDs or those who experience intolerable adverse effects from alternative therapies (Conaghan et al., 2019; Klotz, 2012). Meta-analytical evidence has demonstrated limited benefit over placebo in reducing OA pain, particularly in knee and hip OA, and its use is increasingly de-emphasized in clinical guidelines (Bannuru et al., 2019; Fr et al., 2024).

**TABLE 2:** Pharmacological therapies for osteoarthritis: clinical use, evidence, and limitations.

Therapeutic class	Agent(s)	Clinical use	Efficacy (evidence level)	Limitations/safety profile	References
Topical NSAIDs	Diclofenac gel 1%–2%	First-line for localized pain (knee, hand); especially in older adults or those with comorbidities	High: Comparable efficacy to oral NSAIDs for superficial joints; multiple RCTs and meta-analyses^a^	Minimal systemic absorption; local skin reactions in ∼10%	(Derry et al., 2016)
Oral NSAIDs (non-selective and COX-2)	Ibuprofen, Naproxen, Celecoxib	Moderate-to-severe pain in OA; mainstay for symptomatic management	High: Superior analgesic effect vs. placebo and paracetamol in multiple high-quality trials^a^	GI bleeding, renal dysfunction, and increased cardiovascular risk, particularly in long-term use	Bannuru et al. (2019)
Paracetamol (acetaminophen)	Paracetamol	Historically first-line; now reserved for mild cases or as adjunct due to low analgesic efficacy	Low: Minimal benefit over placebo; large-scale meta-analyses show limited functional improvement^b^	Hepatotoxic at high doses; often used despite weak evidence	Machado et al. (2015)
Intra-articular corticosteroids	Triamcinolone acetonide, Methylprednisolone	Short-term relief in moderate-to-severe knee OA with effusion or flare-up	Moderate: Good short-term pain relief (1–3 weeks); no long-term structural benefit^c^	Not for frequent use; risk of cartilage damage, infection, and systemic effects if repeated	McAlindon et al. (2017)
Intra-articular hyaluronic acid	Hylan G-F 20, Sodium hyaluronate	Alternative when NSAIDs are contraindicated; debated effectiveness	Low to Moderate: Conflicting trial outcomes; benefit seen in select populations (e.g., younger, mild OA) ^b^	High cost, not routinely recommended by NICE/OARSI; local adverse events possible	Bannuru et al. (2015)
Duloxetine (SNRI)	Duloxetine	Adjunct in chronic OA pain, especially with central sensitization or comorbid depression	Moderate: Demonstrated improvement in pain and function in RCTs; endorsed in knee OA by OARSI^b^	Side effects include nausea, insomnia, fatigue; cautious use in polypharmacy	Chappell et al. (2011)
SYSADOAs (Glucosamine, Chondroitin)	Glucosamine sulfate, Chondroitin sulfate	Controversial; used in early OA or as an adjunct	Low to Moderate: Chondroitin may be effective in hand OA; glucosamine benefit is inconsistent^b^	Slow onset; quality and bioavailability vary; not universally recommended	Wandel et al. (2010)
Emerging Biologic Therapies	Tanezumab (anti-NGF monoclonal antibody)	Investigational; for severe, treatment-resistant OA pain	Moderate to High: Significant analgesic effect in trials; development halted due to adverse joint events^c^	Rapidly progressive OA risk; under regulatory evaluation	Schnitzer et al. (2019)

^a^ High-quality evidence from consistent RCTs or meta-analyses.

^b^ Low-quality or conflicting evidence from observational studies or low-powered trials.

^c^ Moderate-quality evidence from RCTs with some inconsistency.

Recent preclinical studies have identified two promising pharmacological strategies for OA management. Senolytics, which are agents that selectively eliminate senescent cells, and ferroptosis inhibitors, which target iron-dependent cell death pathways implicated in cartilage degeneration, are emerging as potential disease-modifying therapies. Senescent chondrocytes accumulate in osteoarthritic joints, contributing to inflammation and tissue breakdown *via* the senescence-associated secretory phenotype (SASP). Preclinical models have shown that senolytic compounds like *navitoclax* or flavonoid-based combinations (e.g., fisetin) can reduce cartilage degradation and synovial inflammation by clearing senescent cells (Fernandez-Moreno et al., 2025; de Magalhães, 2025).Additionally, the combination of senolytics with autophagy enhancers or anti-inflammatory agents has shown synergistic effects on OA symptom relief and structural repair (Garcia-Dominguez et al., 2025).Parallel advances have emerged in ferroptosis research, revealing that this iron-dependent oxidative cell death plays a key role in chondrocyte dysfunction in OA. Several natural compounds and small molecules, such as tetrandrine, proanthocyanidins, sodium tanshinone IIA sulfonate, and dehydrotanshinone II A,have been demonstrated to inhibit ferroptosis, reduce ROS levels, and preserve cartilage matrix in OA animal models (Xing et al., 2025; Xu M. et al., 2025; Guan et al., 2025). Notably, CDO1, a key regulator of cellular redox balance, has been identified as a ferroptosis-related therapeutic target in OA *via* integrated multi-omics analyses (Zhao et al., 2025). Moreover, multi-targeted nanoparticle systems combining ferroptosis inhibition with anti-inflammatory or antioxidant therapy show promise in enhancing drug delivery and therapeutic efficacy (Li et al., 2026).

#### 3.2.2 Intra-articular therapies

Intra-articular (IA) therapies represent a key component of localized pharmacologic management in OA, particularly for patients who exhibit inadequate response to oral medications or are contraindicated for systemic therapy. These interventions aim to deliver targeted symptom relief directly within the joint space, with the principal agents including corticosteroids, hyaluronic acid (HA), and emerging biologics such as platelet-rich plasma (PRP) and cell-based products (Wehling et al., 2017; Testa et al., 2021). IA corticosteroids (e.g., methylprednisolone, triamcinolone) are among the most frequently used IA agent in OA treatment. Their anti-inflammatory action is mediated through inhibition of pro-inflammatory cytokines and leukocyte trafficking within the synovium. Clinical evidence supports their efficacy in providing short-term pain relief (3–6 weeks) and functional improvement, especially in patients with inflammatory phenotypes of OA or joint effusions (Iannitti et al., 2011; Paik et al., 2019; Najm et al., 2021). However, repeated or frequent injections may pose risks, including chondrotoxicity and acceleration of cartilage degeneration. As such, their use is generally limited to a few administrations per year per joint (Bensa et al., 2024; Guermazi et al., 2023). HA is a major component of synovial fluid, serving a structural and lubricating function within the joint. In OA, both the molecular weight and concentration of endogenous HA are reduced, contributing to impaired viscoelasticity and increased mechanical stress on cartilage. IA administration of exogenous HA aims to restore synovial fluid rheology, improve joint mechanics, and potentially exert chondroprotective effects (Tamer, 2013; Gupta et al., 2019). Clinical outcomes from HA therapy remain variable. Some randomized controlled trials report modest improvements in pain and function, particularly in patients with mild-to-moderate knee OA, while others show minimal benefit compared to placebo (Chavda et al., 2022; Lana et al., 2016). A 2023 OARSI consensus report concluded that HA may offer limited but clinically relevant benefit in select patients, though guideline recommendations remain inconsistent due to heterogeneity in formulations and trial quality (Macri et al., 2023). Furthermore, PRP has garnered increasing attention due to its autologous origin and potential regenerative properties. Rich in growth factors such as platelet-derived growth factor (PDGF), transforming growth factor-β (TGF-β), and insulin-like growth factor-1 (IGF-1), PRP may promote tissue repair and modulate synovial inflammation (Gato-Calvo et al., 2019). Bensa et al. demonstrated that PRP injections provide statistically and clinically significant improvements in both pain and function in patients with knee OA. Their meta-analysis of 18 randomized controlled trials involving 1995 patients showed that PRP significantly outperformed placebo at 1, 3, 6, and 12 months follow-up, with the greatest clinical relevance observed at 3 and 6 months. Notably, PRP preparations with higher platelet concentrations were associated with superior outcomes, suggesting that platelet dose is a key determinant of therapeutic efficacy in intra-articular PRP administration for knee OA (Bensa et al., 2024). In addition, Adrien et al. found that PRP injections, when combined with a structured rehabilitation program, led to clinically significant and sustained improvements in pain and joint function in patients with large joint OA. Their retrospective analysis of 252 patients demonstrated a 49% reduction in pain (VAS) and a 44% improvement in function (SANE) at 6 months, with benefits maintained at 12 months. Moreover, multiple PRP sessions and higher levels of sports activity, particularly at the competitive level, were associated with more favorable outcomes, suggesting a dose-response relationship and the influence of biomechanical conditioning on PRP efficacy (Schwitzguébel et al., 2025). However, variation in PRP preparation methods, injection protocols, and lack of standardization have limited widespread adoption and regulatory endorsement. Other IA, including mesenchymal stem cells (MSCs), autologous protein solutions, and liposomal corticosteroids, are under investigation for their potential to modify disease progression. While early-phase studies have demonstrated safety and some efficacy signals, larger randomized trials are necessary to validate their long-term effects, cost-effectiveness, and comparative utility (Rodríguez-Merchán, 2022; Cao M. et al., 2025; Hunter et al., 2022).Hernigou et al. found that subchondral injection of bone marrow-derived MSCs is significantly more effective than intra-articular delivery in delaying total knee arthroplasty (TKA), with a 15-year randomized trial showing a 70% TKA rate in knees treated intra-articularly *versus* only 20% in those treated subchondrally (Hernigou et al., 2021a). In a separate study comparing subchondral MSC injection to contralateral TKA in elderly patients, MSC therapy provided comparable long-term pain relief and functional outcomes, with only 18% of MSC-treated knees requiring later arthroplasty. Notably, persistent subchondral bone marrow lesions were predictive of eventual TKA, underscoring their role as imaging biomarkers of therapeutic response (Hernigou et al., 2021b).

In contrast, a randomized controlled trial by Nouri et al. compared the efficacy of IA injections of PRP, HA, and a combination of both in hip OA. While all groups showed some improvement in pain and function at 2 and 6 months post-injection, the outcomes were modest and not significantly different in terms of long-term structural benefit. The combination therapy showed slightly better scores in WOMAC and Lequesne indices than HA alone, but not enough to support strong clinical superiority (Nouri et al., 2022). Similarly, another randomized study by Ghorbani et al. involving patients with knee OA reported that PRP injections were more effective than HA in reducing pain and stiffness scores. However, the clinical benefit remained confined to the short term. At the 5-month follow-up, although both groups improved, the magnitude of improvement was moderate and did not lead to complete functional recovery. Moreover, despite statistical significance, the improvements did not reach minimal clinically important difference (MCID) thresholds for all patients (Ghorbani et al., 2024). These findings align with broader systematic reviews and meta-analyses which suggest that while IA PRP and HA may offer symptomatic relief in early-stage OA, their impact on disease progression, cartilage regeneration, or long-term pain control remains questionable. The variability in product preparation, lack of standardization, and placebo effects further cloud their clinical utility (Tschopp et al., 2023), (Paget et al., 2023).

### 3.3 Surgical intervention

Surgical management of OA is typically reserved for individuals with advanced disease who have not responded to conservative measures, including pharmacologic and non-pharmacologic therapies. The primary goal of surgical intervention is to alleviate pain, restore joint function, and improve quality of life. The choice of procedure is guided by the joint involved, severity of structural damage, patient age, activity level, and comorbidities (Table 3) (Katz et al., 2010; Brumat et al., 2022).

**TABLE 3:** Comparative outcomes of surgical interventions in osteoarthritis.

Intervention	Indication	Common joints	Short-term outcomes	Long-term outcomes	Complications	Patient selection considerations
Arthroscopic debridement	Mechanical symptoms (select cases)	Knee	Minimal benefit	No long-term efficacy	Infection, thromboembolism	Not recommended in the absence of mechanical locking
High tibial osteotomy (HTO)	Unicompartmental knee OA	Knee	Pain relief, load redistribution	Delay in need for TKR	Over or undercorrection	Ideal for younger, active patients
Total joint replacement (TKR, THR)	End-stage OA, failed conservative management	Knee, Hip	Significant pain relief	Durable for 15–20 years	Infection, loosening, stiffness	Requires shared decision-making
Joint fusion (arthrodesis)	End-stage OA in low-demand joints	Ankle, wrist, thumb CMC	Pain relief	Functional loss in the fused joint	Adjacent joint degeneration	Considered when motion preservation is less critical
Interpositional arthroplasty	Hand OA (thumb base)	Thumb CMC	Motion preserved	Limited evidence	Instability, implant failure	Used selectively in hand surgery

#### 3.3.1 Autologous chondrocyte implantation (ACI)

ACI is a cell-based surgical technique designed to repair focal articular cartilage defects, particularly in the knee, to restore hyaline-like cartilage and delay OA progression. The procedure involves harvesting a small sample of healthy cartilage from a non-weight-bearing area, isolating and expanding chondrocytes *ex vivo*, and implanting them into the cartilage defect, often under a periosteal flap or collagen membrane (Welch et al., 2016; Karuppal, 2017). ACI is primarily indicated for younger, active patients with isolated, symptomatic full-thickness defects larger than 2–2.5 cm^2^, and is generally not recommended in advanced OA with diffuse degeneration (Minas et al., 2010; Jones and Peterson, 2006).

Clinical success with ACI is highly dependent on defect etiology, morphology, and location. Traumatic chondral lesions, such as those from sports injuries,are among the most responsive to ACI due to their well-circumscribed borders and otherwise healthy surrounding cartilage matrix, which provides a favorable environment for graft integration (Rodríguez-Merchán et al., 2012; Mithoefer et al., 2011). Similarly, early-stage focal osteoarthritic lesions, where degenerative changes are limited and biomechanical alignment is preserved, can also benefit from ACI,though outcomes are generally less predictable compared to purely traumatic defects (Brittberg et al., 2016; Kurz et al., 2023). Importantly, diffuse osteoarthritis, characterized by widespread cartilage thinning, subchondral bone sclerosis, and inflammatory milieu, is a contraindication for ACI due to poor regenerative potential and high failure rates (Muthu et al., 2023; Liu et al., 2022).

Anatomical defect location plays a critical role in outcome predictability. The medial femoral condyle is the most commonly treated and tends to yield better outcomes compared to the patellofemoral compartment, which presents with complex biomechanics and greater shear forces during flexion (Ebert et al., 2024; Trattnig et al., 2011). A meta-analysis by Mithoefer et al. demonstrated that medial condylar defects had significantly higher return-to-activity rates and more durable repair compared to patellar lesions (Mithoefer et al., 2011). Additionally, defect size and containment are key determinants,well-contained lesions >2 cm^2^ show better matrix fill and integration, while uncontained or “kissing lesions” have shown less favorable histological and clinical results (Brittberg et al., 2016; Choe et al., 2022). Recent advances, such as atrix-assisted autologous chondrocyte implantation (MACI), have improved surgical handling and defect conformity, especially in irregular or hard-to-access locations, by providing a pre-seeded scaffold that promotes even chondrocyte distribution and better ECM deposition (Zhou et al., 2024).

Furthermore, radiological and histological evaluations underscore the importance of defect characterization. MRI-based biomarkers, such as T2 mapping and dGEMRIC, can identify early OA and assess repair quality over time, aiding both in patient selection and long-term monitoring (Trattnig et al., 2011; Zhang et al., 2023). In the context of early osteoarthritis, recent trials suggest that MACI may slow degenerative progression when applied to isolated focal areas, though longitudinal studies remain limited and are complicated by variable definitions of early OA across trials (Muthu et al., 2023). Ultimately, clear distinction between focal cartilage injury and diffuse degenerative joint disease is essential to determine ACI eligibility, with emerging imaging, biomaterials, and scaffold innovations contributing to expanding its therapeutic window.

#### 3.3.2 Arthroscopy

Arthroscopic debridement and lavage were historically used in the management of knee OA, especially in the presence of mechanical symptoms such as locking or catching (Medical Advisory Secretariat, 2005). However, several large randomized controlled trials, including the pivotal study by Moseley et al., and subsequent meta-analyses have demonstrated no long-term benefit over placebo or conservative therapy in terms of pain, function, or disease progression (Moseley et al., 2002). As a result, contemporary guidelines, including those from the American Academy of Orthopaedic Surgeons (AAOS) and OARSI, strongly advise against routine arthroscopic surgery for degenerative OA in the absence of clear mechanical indications such as unstable meniscal tears or loose bodies (Moseley et al., 2002; Brophy and Lowry, 2023).

#### 3.3.3 Osteotomy (joint preservation strategy)

Osteotomy, particularly high tibial osteotomy (HTO) for varus-aligned medial compartment knee OA, is a joint-preserving surgical option primarily indicated in younger, active patients (<60 years) with unicompartmental disease and intact lateral joint surfaces (Murray et al., 2021). The procedure involves realigning the mechanical axis of the limb to offload the diseased compartment, thereby redistributing joint stress and delaying the need for total knee arthroplasty (Peng H. et al., 2021; Ferrera and Menetrey, 2022). Clinical studies report significant improvements in pain and function, with survivorship rates of up to 75%–85% at 10 years postoperatively (Mustamsir et al., 2025; Ollivier et al., 2021). Lateral distal femoral osteotomy (LDFO) is similarly employed for valgus-aligned lateral compartment disease, though with more limited long-term data. Key predictors of success include preoperative alignment correction, joint congruency, and preservation of joint space width (Cameron et al., 2015; O'Malley et al., 2016). In parallel, joint distraction has emerged as another joint-preserving approach, particularly for patients with end-stage OA who are otherwise candidates for arthroplasty. Unlike osteotomy, joint distraction does not rely on realignment but instead involves temporary mechanical separation of the joint surfaces using an external fixation device. This unloading period promotes cartilage regeneration and modifies the subchondral bone environment (Shtroblia et al., 2025). A study has shown that distraction can lead to symptomatic relief and radiographic evidence of cartilage repair, with some studies demonstrating benefits lasting up to 10 years (Jansen et al., 2022). Mechanistically, the benefits are thought to arise from partial unloading, synovial fluid pressure oscillation, enhanced activity of mesenchymal stem cells, and modulation of inflammatory and reparative pathways within the joint (Jansen et al., 2022). As with osteotomy, patient selection is critical, and this modality is especially suited for relatively young, active individuals in whom delaying prosthetic joint replacement is a priority (McGonagle et al., 2017).

#### 3.3.4 Total joint arthroplasty (TJA)

TJA, particularly TKA and total hip arthroplasty (THA), represents the most effective and durable surgical treatment for end-stage OA. These procedures consistently demonstrate high levels of pain relief, restoration of function, and improved health-related quality of life (HRQoL), with implant survival exceeding 90% at 15–20 years in well-selected patients (Feng et al., 2018; Pollock et al., 2016; Madry, 2022). THA is considered among the most successful surgical procedures in all of medicine, with patient satisfaction rates surpassing 95% (Serfaty, 2020; Shon et al., 2019). Advances in implant technology (e.g., highly cross-linked polyethylene, cementless fixation), computer-assisted navigation, and perioperative care protocols (e.g., enhanced recovery after surgery [ERAS]) have improved short- and long-term outcomes while reducing complications and hospitalization times (Min et al., 2020; Altman et al., 2019). However, the risk of surgical site infection, thromboembolism, aseptic loosening, and periprosthetic fracture must be carefully weighed against the anticipated benefit, particularly in elderly or frail patients (Antonelli and Chen, 2019; Li et al., 2022). Moreover, pain relief after TKA is not assured. Wylde et al. emphasize that chronic pain after TKA affects up to 34% of patients and significantly impacts quality of life, often persisting for months or years postoperatively despite technically successful surgeries (Wylde et al., 2018). Beswick et al. systematically reviewed prospective studies and found that 9%–20% of patients continue to experience moderate to severe pain after THA or AKA, underscoring a critical need to identify predictors and improve pain management strategies. However, a 2025 review by Trojian and Naik emphasized that TKA and THA remain the gold standard interventions for patients with severe hip or knee OA who do not respond to non-operative therapy, with evidence supporting significant pain reduction and sustained functional improvement across large cohort studies and registry data (Trojian and Naik, 2025).

#### 3.3.5 Unicompartmental knee arthroplasty (UKA)

UKA arthroplasty is indicated in patients with isolated medial or lateral compartment OA and intact cruciate ligaments. Compared to TKA, UKA offers the advantage of a smaller incision, faster recovery, and preservation of native joint kinematics (Mittal et al., 2020; Rodríguez-Merchán and Gómez-Cardero, 2018). However, registry data show a higher revision rate for UKA than TKA, particularly in younger and more active patients. Careful patient selection and surgical precision are essential for optimal outcomes. Long-term studies have shown survivorship rates of approximately 80%–90% at 10 years (Rodríguez-Merchán and Gómez-Cardero, 2018; Deschamps et al., 2012).

## 4 Prevention

### 4.1 Weight management and obesity prevention

Obesity is a major modifiable risk factor for OA, particularly affecting weight-bearing joints such as the knees. Excess adipose tissue contributes to increased mechanical loading and to systemic inflammation *via* adipokine dysregulation. Miao et al. found that high body mass index (BMI) significantly correlates with both global and regional OA burden, reinforcing weight management as a central preventive strategy (Miao et al., 2025). Emerging evidence also highlights the role of lean body mass (LBM) in joint health. A large-scale NHANES-based study (n = 31,172) identified a nonlinear inverse association between LBM and OA risk, with a protective threshold at 52.26 kg (Lu H. et al., 2025). Lower LBM, often seen in obesity-related sarcopenia, was associated with greater OA prevalence, underscoring the importance of preserving muscle mass to maintain joint stability and reduce mechanical load (Lu H. et al., 2025). In addition, myokines, particularly irisin, a hormone released during muscle contraction, have been shown to exert anti-inflammatory and chondroprotective effects. Irisin has been found to enhance collagen synthesis, reduce oxidative stress, and inhibit MMP activity, thereby supporting cartilage integrity (Ning et al., 2022; Wang F. S. et al., 2020). However, weight loss programs should prioritize preservation of LBM, using resistance training and adequate protein intake, to optimize joint support and stimulate beneficial myokine responses (Olson and Eccleston, 2024; Messier et al., 2018).

### 4.2 Joint protection and injury prevention

**FIGURE 4:**
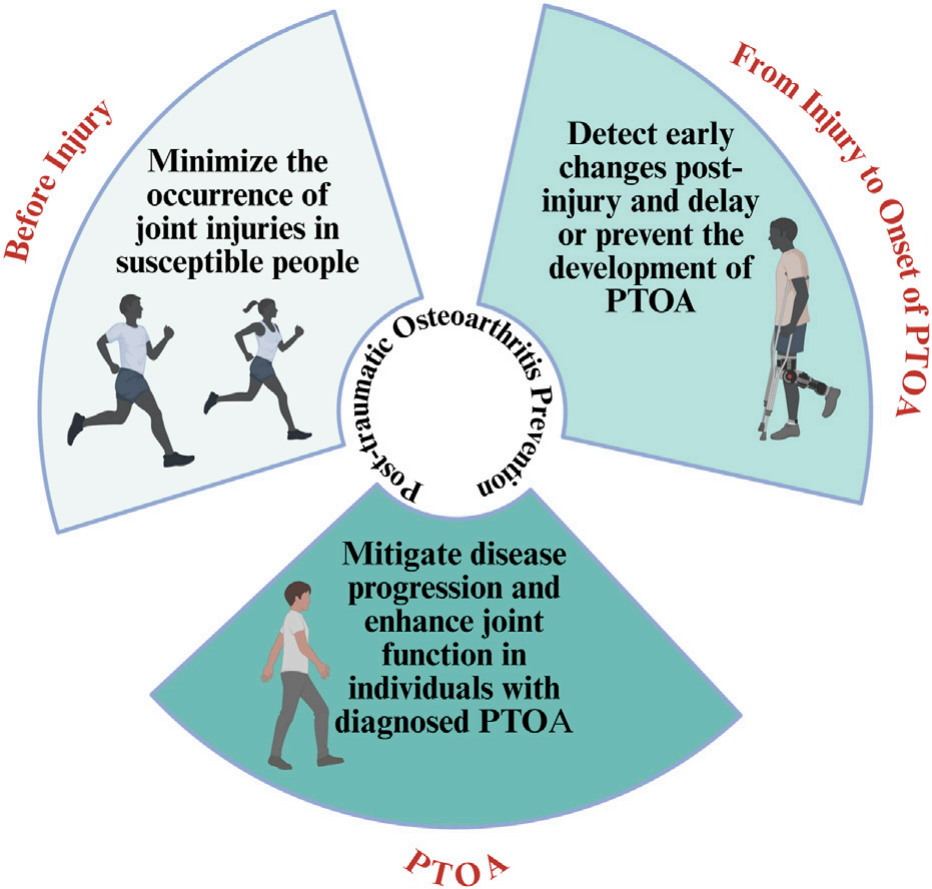
Prevention strategies for post-traumatic osteoarthritis (PTOA).

Trauma to intra-articular structures such as the ACL, meniscus, and articular cartilage, is a well-established risk factor for the development of post-traumatic osteoarthritis (PTOA) (Wang L. J. et al., 2020). It is estimated that approximately 50% of individuals who sustain major joint injuries, such as ACL ruptures, will develop radiographic OA within 10–15 years, even following surgical reconstruction. These injuries disrupt joint biomechanics, induce inflammatory cascades, and initiate cartilage degradation, all of which contribute to the early onset of OA, often in younger, otherwise healthy individuals (Carbone and Rodeo, 2017; Kraus and Hsueh, 2024). Primary prevention of joint injuries is a critical public health strategy, especially in youth and amateur sports settings. Neuromuscular and plyometric training programs (e.g., FIFA 11+, PEP) have demonstrated efficacy in reducing ACL injury risk by enhancing dynamic alignment, balance, and movement control (Viswanathan et al., 2025; Emery and Pasanen, 2019). Systematic implementation of such protocols across athletic populations has shown a 40%–60% reduction in non-contact lower limb injuries, suggesting a potential long-term effect on OA prevention at the population level (Ding et al., 2022; Stephenson et al., 2021). In occupational settings, repetitive joint loading, heavy lifting, kneeling, and squatting have all been linked to increased OA risk, particularly in the knees and hips (Fransen et al., 2011). Ergonomic interventions, job redesign, and protective equipment use (e.g., knee pads) can reduce joint strain and cumulative trauma. Public health policies that enforce workplace standards and promote injury surveillance may contribute to reducing OA burden across physically demanding professions (Hoe et al., 2018; Wang X. et al., 2020). Post-injury secondary prevention is equally critical. Early identification and comprehensive rehabilitation following joint trauma are essential to restore function and minimize risk of PTOA. Strategies such as load management, progressive strengthening, proprioceptive retraining, and regular follow-up imaging can delay or prevent OA progression in high-risk individuals (Figure 4) (Holm-Jensen et al., 2025; Whittaker et al., 2022).

Recent research has also implicated the gut microbiome in OA pathogenesis. Dysbiosis, alterations in gut microbial composition, has been linked to systemic low-grade inflammation, elevated lipopolysaccharides (LPS), and increased cartilage catabolism *via* immune modulation (Boer et al., 2019). In animal models, transplantation of microbiota from obese donors exacerbated cartilage damage, while prebiotic and probiotic interventions were shown to modulate systemic inflammation and improve joint integrity (Shock et al., 2021; Cao B. et al., 2025; Collins et al., 2021). This suggests that obesity-associated OA may in part be mediated by microbiota-derived inflammatory mediators, highlighting gut health as a novel preventive target in metabolic OA. Moreover, dietary omega-3 polyunsaturated fatty acids (n-3 PUFAs), such as EPA and DHA, have shown chondroprotective and anti-inflammatory effects. Omega-3s suppress the NF-κB pathway, reduce the production of pro-inflammatory cytokines (IL-1β, TNF-α), and inhibit MMP-13 expression in cartilage cells (Chen F. et al., 2024; Kar et al., 2023). In both clinical and preclinical studies, higher omega-3 intake has been associated with slower OA progression, reduced synovitis, and improved cartilage volume. Importantly, dietary omega-3s may also modulate the gut microbiome, creating synergistic effects for inflammation reduction (Shawl et al., 2024; Huang et al., 2024).

### 4.3 Physical activity and muscle strengthening

Regular, moderate-intensity physical activity is protective against OA by enhancing joint lubrication, improving muscle support, and reducing inflammation. Exercise programs focused on quadriceps strengthening, proprioception, and balance training can delay or prevent the onset of symptomatic OA, particularly in individuals with early cartilage changes or biomechanical abnormalities. Telerehabilitation approaches are also emerging as scalable tools to deliver preventive care remotely (Plavoukou et al., 2025).

## 5 Future direction

**FIGURE 5:**
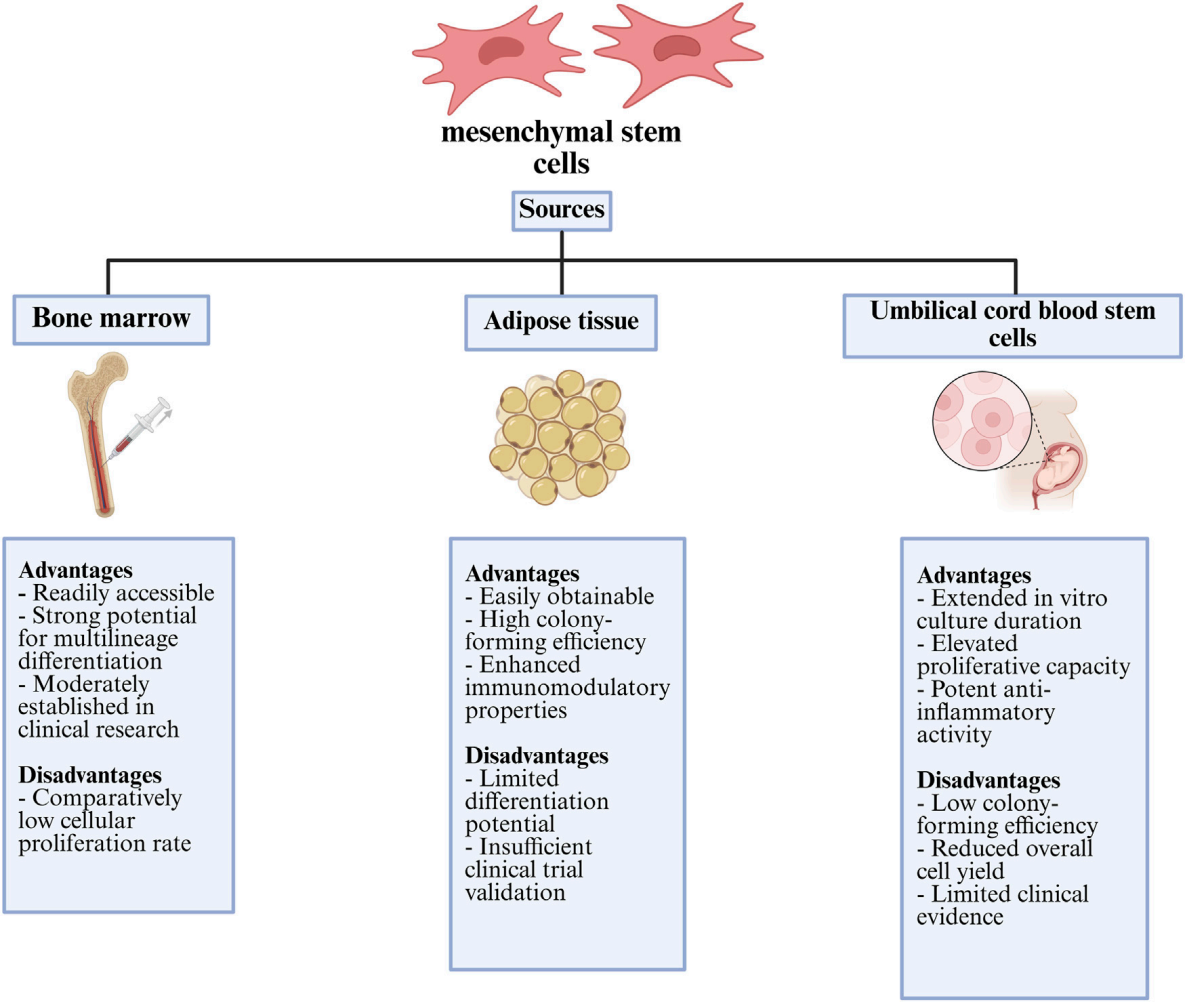
Comparative summary of the main advantages and limitations of mesenchymal stem cells from different tissue origins with clinical and regenerative use.

The landscape of OA research is rapidly evolving from symptom-based management to a deeper understanding of the molecular, biomechanical, and systemic drivers of disease progression. This paradigm shift is propelling innovation across diagnostics, therapeutics, and patient care delivery, with an emphasis on precision medicine, regenerative approaches, and digital health integration (Grässel et al., 2021; Mobasheri et al., 2019). A promising avenue in OA research is the development of precision medicine frameworks, leveraging molecular profiling to stratify patients based on inflammatory, metabolic, or mechanical phenotypes. The integration of genomics, proteomics, and metabolomics, alongside advanced imaging biomarkers, can enable earlier diagnosis, individualized treatment selection, and more accurate monitoring of disease progression (Zhai, 2021; Veillette et al., 2015). For instance, Lu et al. emphasize the need to develop and validate molecular signatures that predict structural worsening and therapeutic response in OA, which could guide tailored interventions at earlier stages of the disease (Lu J. S. et al., 2025). Recent imaging advances also enhance the biomarker-driven approach. Quantitative MRI techniques, including T2 mapping and dGEMRIC, can noninvasively assess early cartilage degeneration. Gao et al. developed a “digital twin” of the knee using quantitative MRI data and AI-driven modeling to forecast disease progression and support precision interventions (Hoyer et al., 2025). On the genomic front, several single-nucleotide polymorphisms (SNPs) have been associated with OA susceptibility and progression, including variants in the GDF5, SMAD3, and MMP13 genes. These markers hold potential for predictive modeling and risk stratification (Waheed and Rai, 2024; Wang et al., 2016; Hong et al., 2018). A recent study by Pan also underscores the role of Mendelian randomization in confirming causal relationships between biomarker profiles and OA outcomes, reinforcing the biological relevance of circulating factors (Pan, 2025). Furthermore, synovial tissue proteomics and RNA sequencing are being applied to detect local biomarkers reflective of active joint inflammation, enabling more accurate subtyping of inflammatory OA phenotypes (Carr et al., 2020). Patient phenotyping is increasingly recognized as a pivotal factor in optimizing future OA clinical trials. OA encompasses diverse phenotypes, including inflammatory, metabolic, mechanical, and post-traumatic subtypes, each driven by distinct pathophysiologic mechanisms (Saxer et al., 2024). Applying a “one-size-fits-all” approach in past DMOAD trials likely diluted therapeutic effects and contributed to negative outcomes. To address this, modern clinical trial designs are incorporating biomarker-guided stratification and multi-omics profiling to enrich study populations with patients most likely to respond to specific interventions (Schäfer and Grässel, 2022; Kim et al., 2022). For example, patients with synovitis may benefit more from anti-inflammatory biologics, while those with biomechanical OA may respond better to structural modulators or orthobiologics (Knights et al., 2023; Kl and oppenburg, 2024). Regulatory authorities are increasingly supportive of these strategies, recognizing that precision trial enrollment can enhance efficacy signals, reduce variability, and align therapeutic outcomes with specific disease subtypes. In this evolving paradigm, phenotype-driven research is no longer optional, it is essential for regulatory approval, clinical impact, and the successful translation of next-generation OA therapies (Chu et al., 2025; Edwards et al., 2016). Furthermore, Chen et al. identified dipeptidyl peptidase-4 (DPP-4) as a candidate biomarker linked to chondrocyte catabolism and synovial activation, suggesting a novel target for endotype-specific intervention (Chen et al., 2025). Ge et al. conducted a comprehensive multi-omics analysis of synovial tissue and fluid in knee OA and identified distinct metabolic and proteomic profiles associated with disease progression, highlighting key roles for fibronectin 1 (FN1), TGFBI, and the tricarboxylic acid cycle (TCA) in synovial osteogenesis. Their findings suggest that alterations in collagen metabolism, ECM remodeling, and energy metabolism serve as mechanistic drivers and offer a set of candidate biomarkers for diagnostic and therapeutic targeting (Ge et al., 2025). In a pivotal study, Huo et al. identified a panel of seven m7G-related hub genes, including SNUPN, RNMT, NUDT1, LSM1, LARP1, CYFIP2, and CYFIP1, that were differentially expressed in OA synovial tissue compared to healthy controls. Using integrated computational modeling and validation *via* RT-qPCR, these genes were incorporated into a diagnostic risk model with high predictive accuracy (AUC >0.9). Their functional enrichment analyses linked these genes to mRNA metabolic processes, immune cell regulation, and RNA transport pathways, confirming their potential role in OA pathogenesis and immune-mediated joint degradation (Huo et al., 2025). Recent targeted therapies are focusing on molecular pathways implicated in OA progression, including TGF-β/ALK5 signaling, Wnt/β-catenin modulation, NF-κB suppression, and ferroptosis inhibition (Tian et al., 2025; Hadzic and Beier, 2023). Agents that block ADAMTS-5, neutralize nerve growth factor, or reduce cellular senescence have shown disease-modifying potential (Jiang et al., 2021). Moreover, significant progress has been made in biological therapies, particularly engineered cytokine modulators, macrophage-reprogramming strategies, and extracellular vesicle-based treatments derived from MSCs, all of which have demonstrated anti-inflammatory, chondroprotective, and immunomodulatory effects (Cotter et al., 2020; Xu G. et al., 2025) (Figure 5). Exosome-based therapeutics, in particular, have shown the ability to deliver microRNAs and regulatory proteins to modify chondrocyte metabolism, reduce synovial fibrosis, and attenuate subchondral angiogenesis (Ni et al., 2020; Yang et al., 2025). Furthermore, advanced drug-delivery systems, including nanoparticle formulations, ROS-responsive hydrogels, thermo-sensitive polymers, and sustained-release microspheres, are also rapidly evolving. These platforms improve intra-articular retention, enhance drug bioavailability, and allow localized release of antioxidants, growth factors, or small-molecule inhibitors precisely within inflamed or mechanically stressed regions of the joint (Yin et al., 2024; Huang H. et al., 2022). In the field of tissue engineering, multifunctional scaffolds integrating bioactive molecules, immunomodulatory cues, and stem cells are emerging as next-generation regenerative platforms (Im, 2018). Recent investigations have shown that composite scaffolds incorporating growth factors (e.g., TGF-β3, BMP-7), gene-delivery vectors, or mechanical-responsive components can simultaneously promote cartilage regeneration, modulate synovial inflammation, and restore osteochondral integrity (Shanto et al., 2025; Liang et al., 2023). Advances in 3D bioprinting enable anatomically accurate osteochondral constructs with zonal organization and gradient stiffness, improving integration and long-term repair quality (Chartrain et al., 2022; Lai et al., 2025), (Turunen et al., 2023). Nevertheless, the pursuit of disease-modifying osteoarthritis drugs (DMOADs) represents another transformative direction (Li S. et al., 2023). While current treatments largely focus on symptom relief, agents such as Wnt pathway modulators (e.g., lorecivivint), ADAMTS-5 inhibitors, and nerve growth factor (NGF) antagonists like tanezumab are under investigation for their potential to alter disease progression (Jiang et al., 2021; Cho et al., 2021; Kou et al., 2023). Although clinical trial outcomes have been mixed, ongoing research aims to optimize patient selection, dosing regimens, and combinatory strategies. Furthermore, novel formulations of intra-articular therapeutics, such as glucosinolates and enhanced hyaluronic acid, are being explored for their potential to provide both analgesic and structural benefits (Gambari et al., 2025).

**FIGURE 6:**
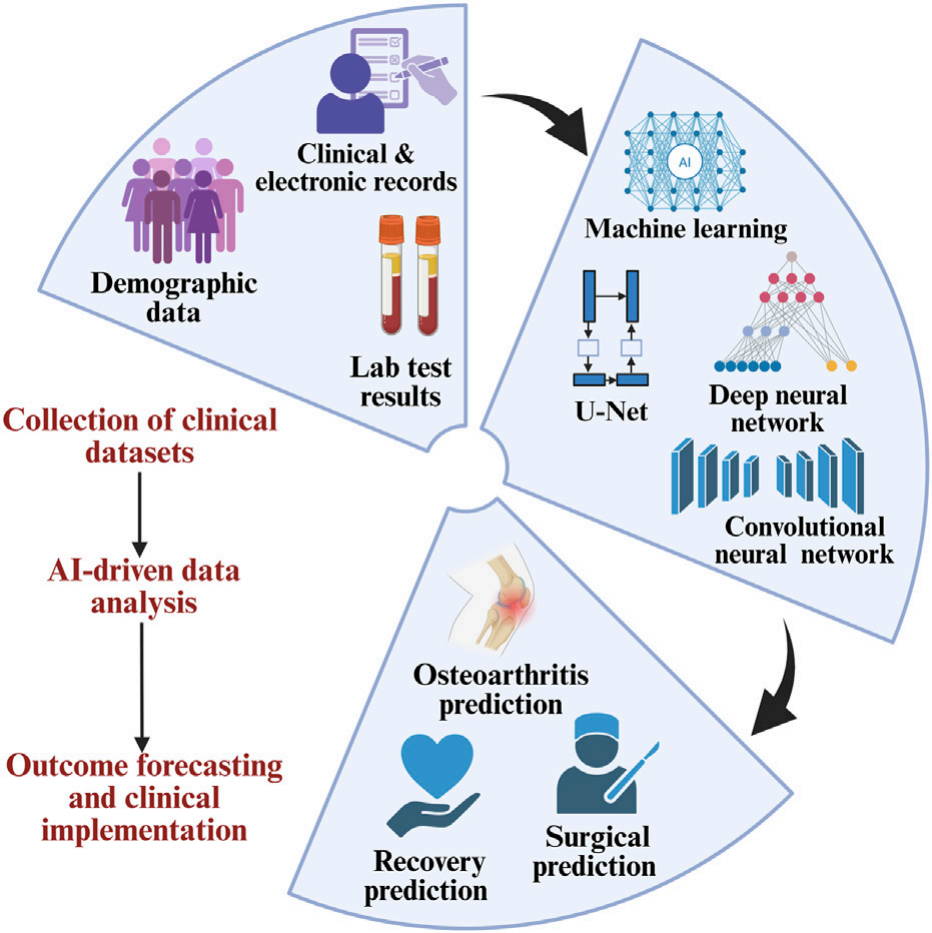
AI-based analysis of clinical data enables the prediction of osteoarthritis outcomes and supports clinical decision-making.

Artificial intelligence (AI) and digital health technologies are increasingly being integrated into OA care. AI-powered image analysis systems can detect subtle cartilage changes and joint space narrowing before they become clinically apparent, offering the possibility of earlier intervention. In the omics domain, AI facilitates the processing and interpretation of complex high-dimensional datasets, which is critical for identifying cell-type-specific gene expression patterns, post-transcriptional regulators, and predictive biomarkers. For instance, Ou et al. describe how transcriptomic and proteomic analyses, enhanced by machine learning classifiers like random forests and support vector machines, have identified OA-relevant genes such as RIPK3, PDK1, and CDH2. These genes are associated with chondrocyte necroptosis, metabolic dysregulation, and immune cell infiltration, offering a more nuanced understanding of disease mechanisms compared to traditional markers like CTX-II or IL-6 (Ou et al., 2025). AI also enables multimodal integration of data sources, including clinical records, electronic health records (EHRs), and laboratory tests, to construct predictive models for disease onset, progression, and surgical outcomes (Figure 6). Notably, predictive models incorporating AI have demonstrated high accuracy in forecasting the need for joint replacement surgery and in stratifying patients based on their expected response to specific interventions (Mohsen et al., 2022; Lee et al., 2022). Wearable devices and mobile health platforms are also emerging as tools for remote monitoring of physical activity, gait abnormalities, and treatment adherence. According to Takase et al., such technologies could play a central role in the real-time assessment and personalization of therapy for OA patients (Takase et al., 2025). Another important emerging concept is the role of mitochondrial dysfunction and oxidative stress in chondrocyte senescence and cartilage degradation. Novel therapies targeting mitochondrial quality control pathways, such as mitophagy enhancers and antioxidant regulators, may represent a new class of interventions capable of modulating OA pathophysiology at the cellular level (Picca et al., 2020; Guo et al., 2025). Wu et al. describe how these mechanisms are increasingly linked to the metabolic-inflammatory axis of OA, offering future therapeutic targets beyond conventional anti-inflammatories (Wu et al., 2025).

The future of OA care is poised to be transformed by innovations in systems biology, biomaterials, digital health, and pharmacology. These advancements underscore the transition from a reactive, symptom-based approach to a proactive, mechanism-driven model of care. Success will depend on collaborative efforts across basic science, clinical research, and health technology sectors to translate these promising developments into safe, effective, and accessible therapies for the global OA population.

## 6 Conclusion

Advances in molecular profiling, imaging, and regenerative strategies are redefining our understanding of osteoarthritis beyond structural degeneration. Bridging basic science with precision therapeutics offers a path toward earlier diagnosis, targeted intervention, and long-term disease modification. Continued interdisciplinary research will be essential to translate these innovations into accessible, patient-centered care.
